# Learned prioritization yields attentional biases through selection history

**DOI:** 10.3758/s13414-020-01970-y

**Published:** 2020-01-23

**Authors:** Jaap Munneke, Jennifer E. Corbett, Erik van der Burg

**Affiliations:** 1 College of Health and Life Sciences, Brunel University London, Kingston Lane, London, UB8 3PH UK; 2 Division of Psychology, Brunel University London, London, UK; 3 Centre for Cognitive Neuroscience, Brunel University London, London, UK; 4 TNO Human Factors, Netherlands Organisation for Applied Scientific Research, Soesterberg, The Netherlands; 5 Brain and Cognition, University of Amsterdam, Amsterdam, The Netherlands; 6 Department of Experimental and Applied Psychology, Vrije Universiteit Amsterdam, Amsterdam, The Netherlands

**Keywords:** Attention: Selective, attentional capture

## Abstract

While numerous studies have provided evidence for selection history as a robust influence on attentional allocation, it is unclear precisely which behavioral factors can result in this form of attentional bias. In the current study, we focus on “learned prioritization” as an underlying mechanism of selection history and its effects on selective attention. We conducted two experiments, each starting with a training phase to ensure that participants learned different stimulus priorities. This was accomplished via a visual search task in which a specific color was consistently more relevant when presented together with another given color. In Experiment 1, one color was always prioritized over another color and inferior to a third color, such that each color had an equal overall priority by the end of the training session. In Experiment 2, the three different colors had unequal priorities at the end of the training session. A subsequent testing phase in which participants had to search for a shape-defined target showed that only stimuli with unequal overall priorities (Experiment 2) affected attentional selection, with increased reaction times when a distractor was presented in a previously high-priority compared with a low-priority color. These results demonstrate that adopting an attentional set where certain stimuli are prioritized over others can result in a lingering attentional bias and further suggest that selection history does not equally operate on all previously selected stimuli. Finally, we propose that findings in value-driven attention studies where high-value and low-value signaling stimuli differentially capture attention may be a result of learned prioritization rather than reward.

Evidence is rapidly accumulating that a stimulus’s history can result in a lingering attentional bias toward that stimulus or its features (for an overview, see Awh, Belopolsky, & Theeuwes, 2012; Theeuwes, 2018). While attentional biases through stimulus history can be observed under varying experimental conditions (e.g., “intertrial priming”: Maljkovic & Nakayama, 1994, 1996; Theeuwes & Van der Burg, 2008, 2013; or “statistical learning”: Ferrante et al., 2018; Wang & Theeuwes, 2018), one of the more robust observations of selection history’s influence on attention can be perceived for stimuli that at some point were “prioritized” over other stimuli. Recent examples of the intimate link between prioritization and selection history come from studies on the topic of value-driven attention, which show that the repeated selection of a value-signaling stimulus exerts a strong influence on attention long after the association between value and the stimulus has been removed (e.g., Anderson, Laurent, & Yantis, 2011b). Here, prioritization of one stimulus over another, through assigning a reward to some, but not other, stimuli, leads to a lingering bias on attention.

In real-world situations, prioritization of information can be established in multiple ways such that prioritization of a particular stimulus is often depending on a person’s current situation. For instance, the same individual may prioritize water over food, food over coffee, and coffee over water, depending on whether she is thirsty, hungry, or tired. However, in contrast to such a changing *situational* priority relationship, stimuli can also have *fixed* priorities in relation to each other. For example, selecting a stimulus that signals threat (e.g., a stimulus associated with pain) will almost always have a higher priority than selecting a nonthreatening stimulus (e.g., a stimulus that is not associated with pain or discomfort; see Preciado, Munneke, & Theeuwes, 2017). Similarly, a high-reward stimulus will most likely always have a higher behavioral priority than a low-reward stimulus. While situational and fixed prioritization are likely the two end points on a priority continuum, the discrepancy between the two may suggest that selection history does not influence attention in a passive and predetermined way, but suggests that its influence may very well be context dependent. One of the main aims of the current manuscript is to explore the link between this sort of context-dependent prioritization and its influence on attention.

The importance of prioritization as a mechanism underlying attentional control has been well established. Theories on attentional control have postulated that selection is the result of overall activity on a spatial priority map, akin to saliency maps widely used to explain attentional guidance (e.g., Borji, Sihite, & Itti, 2013; Itti & Koch, 2000). In this framework, visual input is preattentively filtered and a map is created to represent different bottom-up features (e.g., spatial frequency, orientation), with activation strength as a marker of perceptual saliency. Attention is guided toward the location of stimuli with the highest relative activations, and these stimuli are selected for further processing. A priority map is similar to a saliency map, but incorporates top-down factors, such as the current goals of an observer, as well as a stimulus’s behavioral relevance (or priority; Bisley & Goldberg, 2010; Fecteau & Munoz, 2006) in addition to bottom-up factors. In the context of a priority map, stimuli with the highest activity levels are most strongly prioritized, and attention is guided to these stimuli, which are subsequently selected for further processing. In this integrated priority map, attentional selection and our subsequent visual experience is driven by the interaction between bottom-up and top-down attention as well as attentional biases due to selection history (Awh et al., 2012). Thus, while it can be argued that prioritization modulates activity on the priority map, it is unclear whether and to what extent situational and fixed prioritization do this in a differential manner, and as such have disparate effects on attentional biases through selection history.

A secondary aim of this study is to better understand the precise link between priority-based selection history and value-driven attentional capture. Arguably, selection history as a source of attentional modulation has most prominently been investigated through studies on value-driven attentional capture. The main result of value-driven attentional capture studies suggests that stimuli that previously signaled reward capture attention even when this reward is no longer available to the observer (Anderson, Laurent, and Yantis, 2011a, b; Anderson & Yantis, 2013; Failing & Theeuwes, 2014; Jahfari & Theeuwes, 2017). A typical value-driven capture task consists of two phases: (1) a training phase, in which the participant learns the association between the selection of a feature-defined target stimulus and the reward associated with this feature, and (2) a testing phase, in which reward is no longer available, but where previously rewarded stimuli act as distractors, such that they capture attention due to their previously rewarding nature. Crucial for the role of prioritization, and hence the current study, are the results from a series of value-driven attention studies that suggest that the strength of attentional modulation in the testing task is directly related to the magnitude of the learned reward signal during the training task, with higher rewards leading to larger capture effects compared with stimuli that signaled a lower reward (e.g., Anderson, Laurent, & Yantis, 2011a; Gong & Li, 2014; MacLean & Giesbrecht, 2015). This difference in response between high and low reward is often attributed directly to a higher reward signal having a stronger effect on the priority map’s activation levels than a lower reward signal would have. Hence, the lingering effect on attentional selection is expected to be stronger. However, a different explanation for this observation was brought forward by Grubb and Li (2018), who state that differences in attentional selection by stimuli that previously signaled high versus low reward may be caused by participants prioritizing high-reward stimuli over low-reward stimuli during the training phase to maximize monetary gain (see also Le Pelley, Mitchell, Beesley, George, & Wills, 2016). According to this theory, prioritization effectively creates a top-down set in which target stimuli that are equally relevant to the participant in the reward training task become associated with unequal priorities that carry over into the testing task. In other words, as the features that signal high and low reward remain constant throughout the training task (e.g., Anderson et al., 2011a, b), observers may learn to prioritize the high-reward stimulus feature over the low-reward stimulus feature in the training task, leading to stronger history effects in the testing task for higher prioritized stimuli. As such, observed attentional differences between high and low reward do not necessarily reflect a direct expression of value on selection history, but rather an indirect shift in behavioral prioritization of repeatedly perceived stimuli. An explanation in terms of prioritization, rather than reward signal, is in line with a small set of recent studies that found persistent attentional modulation from previously selected but unrewarded stimuli in a series of tasks highly similar to the default value-driven capture task, but without administering the actual reward (Grubb & Li, 2018; Miranda & Palmer, 2014; Sha & Jiang, 2016).

To summarize, while the act of prioritizing one stimulus over another is essentially the same as employing a top-down set for the prioritized stimulus, it is unknown whether a fixed top-down set has a similar effect on the priority map, as compared with situations in which a top-down set can change from moment to moment (i.e., situational priority). The present study further investigates whether the observed high-reward versus low-reward bias on attention can be explained in terms of prioritization rather than being a direct effect of the availability of reward. To this end, the current study aims to investigate whether adopting a top-down set that unequally prioritizes different stimuli can lead to effects of selection history that mirror the effects obtained by high-reward and low-reward signaling stimuli in value-driven capture tasks. Such an observation would provide evidence that attentional modulation due to selection history can be achieved in the absence of reward, but that reward can potentially modulate the behavioral priority of different stimuli. An explanation in terms of “prioritization” could be instrumental in understanding the discrepant findings concerning the necessity of value in selection history.

## General methods

**Fig. 1:**
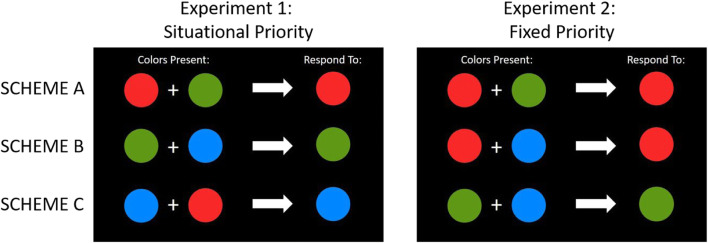
Color-response schemes for Experiments 1 (left) and 2 (right). In Experiment 1, a situational priority relationship was established, such that all three colors had equal overall priorities and were selected equally often over the course of the experiment, but one color was prioritized over the other at the trial level. In Experiment 2, the three colors had a fixed priority relationship throughout the experiment, such that one color was always prioritized over the other two colors (high priority), one color had a lesser priority (low priority), and one color was never prioritized in any case (no priority). (Color figure online)

We conducted two experiments to address the aforementioned questions by manipulating the priority of differently colored stimuli in a training/testing paradigm typically used in studies of value-driven attention (e.g., Anderson et al., 2011a, b). In an initial training task, participants were shown displays of eight shapes (all circles or all diamonds) presented in a circular array. Two of these eight shapes were presented in different colors (out of a total set of three possible colors), with the remaining shapes presented in gray. Prior to the experiment and during a rigorous practice phase, participants were instructed to respond to a target inside one of two colored shapes according to a predefined color-response scheme for the three possible color pairs. In this way, one color-defined stimulus was prioritized over another colored stimulus. The subsequent testing phase was used to investigate lingering attentional biases as a function of selection history in the absence of reward. Experiment 1 tested the effects of prioritized colors with a situational relationship (see Fig. 1, left panel), and Experiment 2 measured prioritization effects with colors that had a fixed relationship (see Fig. 1, right panel). In Experiment 1, a situational priority relationship was established, such that all three colors had equal overall priorities and were selected equally often over the course of the experiment, but one color was prioritized over the other at the trial level. In Experiment 2, the three colors had a fixed priority relationship throughout the experiment, such that one color was always prioritized over the other two colors (high priority), one color had a lesser priority (low priority), and one color was never prioritized in any case (no priority). To foreshadow our results, only stimuli that had a fixed color–priority relationship during the training phase captured attention during the test phase. These effects occurred even though the color and its associated priority were task irrelevant during the test phase. No such effects were found for the color stimuli that were situationally prioritized during the learning phase.

## Experiment 1

Experiment 1 was designed to test how a relationship between two stimuli based on situational priority influences attentional biases due to selection history. In an initial training task, two colored shapes (circles or diamonds) were presented in a circular array consisting of otherwise gray shapes. At the start of the training phase, on-screen instructions were shown explaining which colored circle in each of three possible color pairs contained the target stimulus. The initial training phase was used to further consolidate the learned schemes by having participants repeatedly select the prioritized colored circle and to respond to the target presented inside that stimulus. As illustrated in the left panel of Fig. 1, we established a situational priority relationship by giving Color 1 (e.g., red) a greater priority than Color 2 (e.g., green; Scheme A), giving Color 2 a greater priority than Color 3 (e.g., blue; Scheme B), and giving Color 3 a greater priority than Color 1 (Scheme C). Note that these situational priority schemes in which the overall prioritization of a given color averages out over the three different schemes can be considered one end point of a continuum describing the situational versus fixed relationship between two stimuli. In a subsequent testing phase, we measured the extent to which the situationally prioritized colors biased attention by again presenting a circular array of stimuli with a shape singleton target in one of the colors previously selected in the training phase and one of the other nontarget shapes (the “distractor”) in a different, previously selected color. If situational prioritization occurs at the trial level, then we should observe lingering effects of selection history in the testing task, such that participants respond faster in trials where the target was presented in a color that was prioritized over the distractor color in the training phase, and slower in trials where the distractor color was prioritized over the target color in the training phase. Alternatively, if effects of selection history are solely attributable to prioritization at the task level, then we should not observe any significant differences between response times in the testing task as a function of the training task prioritization of the given target and distractor colors.

### Method

#### Participants

We tested 35 participants with normal or corrected-to-normal vision and no reported history of mental illness. The data of three participants were discarded, as their average accuracy scores on either the training or testing task were more than 2.5 standard deviation below the group mean. Of the 32 remaining participants, 22 were female, and participants had an average age of 21.84 years (*SD* = 4.83 years). All participants gave written, informed consent prior to the experiment. All protocols and procedures were approved by the Vrije Universiteit Amsterdam Ethical Committee, in line with the Declaration of Helsinki.

#### Stimuli and procedure

All participants first completed a training task, during which they learned color–priority associations. This training task was immediately followed by a testing task to measure the extent to which previously prioritized stimuli captured attention (i.e., the effect of behavioral prioritization on selection history). Prior to the start of both tasks, instructions were presented on-screen, and the experimenter verified that participants fully understood the task. Both tasks were conducted in a dimly lit, sound-attenuated room on a standard PC with a 22-in. monitor. Viewing distance was fixed at 75 cm. MATLAB® 2016a and Psychtoolbox 3 (Brainard, 1997; Pelli, 1997) were used for all aspects of stimulus presentation and response collection.

##### Training task

**Fig. 2:**
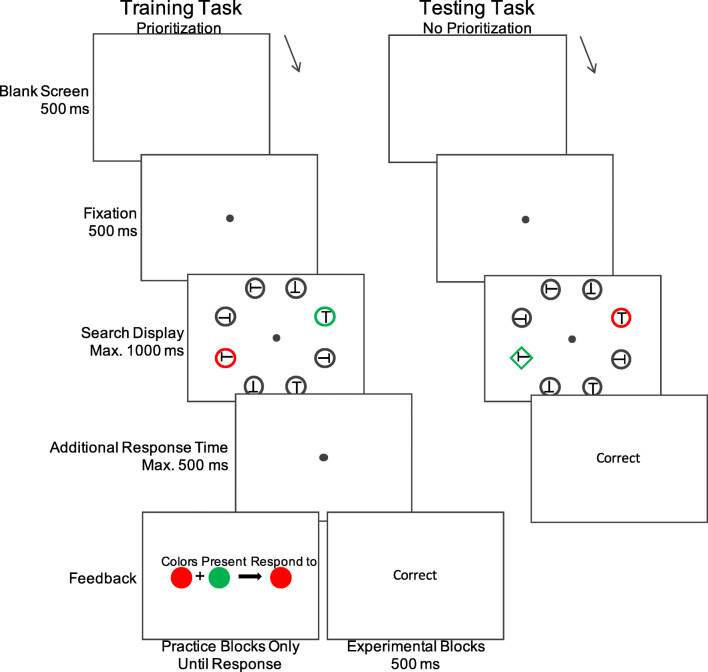
Example trial sequences. In the training task (left), participants responded to the orientation of a “T” (a 4AFC task) presented inside one of two colored shapes (all shapes were either circles or diamonds; circles are presented in this example). The target color was defined by a prelearned color-response scheme (in this example, participants should respond to red when red and green are present). In the testing task (right), participants discriminated the orientation of the “T” inside a colored shape singleton (a green diamond in this example) in the presence of a colored distractor (a red circle in this example). (Color figure online)

The left panel of Fig. 2 illustrates the time course of a trial in the priority training task. After an initial blank screen for 500 ms, a fixation dot (0.2°) appeared for 500 ms, indicating that the trial had started. Participants were instructed to remain centrally fixated throughout the trial. Next, the search display was presented for a maximum duration of 1,000 ms, or until the participant responded. Only responses up to 1,500 ms after the onset of the search display were included in any subsequent analyses. The search display consisted of eight shapes presented around a fixation point on an imaginary circle with a diameter of 5.0°. On a random half the trials, the display consisted of eight circles (diameter: 2.3°). On the other half of trials, the display consisted of eight diamond shapes (diameter ~2.3°). The search display always consisted of six gray outline shapes (11.4 cd/m^2^) and two shapes that were presented in different colors (two colors selected from red, green, or blue; all colors: 27 cd/m^2^). Every shape in the search display contained a gray “T” (11.4 cd/m^2^) that could be rotated 0°, 90°, 180°, or 270°. The target was defined as the “T” shape presented inside one of the two colored shapes, depending on the particular priority schemes the participant was shown at the start of the experiment (described in a subsequent paragraph). All stimuli were presented on a black background. Participants were instructed to respond as quickly and accurately as possible to the orientation of the target “T” using the four arrow keys on the computer keyboard (i.e., a four-alternative forced-choice [4AFC] task). Note that the orientation of all “T”s in the display were randomly chosen with the exception that the “T”s in the two colored shapes were always differently oriented, such that selecting the wrong color for the particular priority scheme always resulted in an incorrect response. After participants responded, accuracy feedback was presented in the center of the screen (“correct”/“incorrect”) for 500 ms. Erroneous responses as well as timed-out trials were considered incorrect in this and all further experiments.

At the start of the experiment, participants were presented with three color-response schemes (see Fig. 1) and were instructed to learn which color to select for each of the three schemes. Importantly, the response schemes were designed so that trial-by-trial priority would average out, and each stimulus would have the same overall priority at the end of the training phase. For example, in Fig. 1 (left panel), red can be considered the high priority color (HPC) when presented together with green, but the low priority color (LPC) when presented with blue. As each color stimulus acted as both an HPC and LPC (depending on the particular scheme), over the course of the training task, each color had the same overall priority. Color schemes were counterbalanced over subjects (either using the schemes illustrated in the left panel of Fig. 2 or the mirror reverse of these schemes) and were presented equally often (each scheme was presented on 33.3% of the trials).

The training phase consisted of eight blocks of 72 trials each (576 trials in total), preceded by two practice blocks of 48 trials each. After each trial in the first practice block, participants received feedback about their accuracy and were presented with an illustration of the color-response scheme defining the target for that trial. Pressing the space bar would start the next trial. In the second practice block, participants again received accuracy feedback after each trial, but the color-response scheme was only presented when the participants responded incorrectly.

##### Testing task

The testing task (see Fig. 2, right panel) was similar to the training task in terms of stimulus presentation and timing, with several notable exceptions. First, the target stimulus containing the “T” shape to respond to was defined as the shape singleton present in each display (i.e., a diamond among circles, or a circle among diamonds), such that the color of any given shape was now task irrelevant. Nonetheless, two colors were always present in the display. One of the colored items in each display was always the shape singleton containing the target, making the other colored shape a salient distractor. The same color schemes participants learned in the training task were used in the testing task. Crucially, on a random half of the trials in the testing task, the target shape singleton was presented in the color that was prioritized (at the trial level) in the training task. On the other half of the testing trials, the distractor shape was presented in the prioritized color. The testing task consisted of two blocks of 96 trials each, preceded by a practice block of 48 trials.

### Results

#### Priority training task

We began our analysis by confirming that priority averaged out over the course of the training task, such that there were no overall differences in response times between the three different priority schemes. We only analyzed nonpractice trials with correct responses (19.31% of total trials discarded). Furthermore, we normalized the response-time data by trimming trials with correct response times that were 2.5 standard deviations above the conditional mean response time (calculated separately for each participant and color scheme), or trials in which the response was faster than 200 ms (1.20% of total trials discarded). All further reaction time analyses are based on the mean response times obtained for each participant and condition. A repeated-measures ANOVA on the mean response times, with color scheme (Scheme A, B, or C) as the only within-subjects factor, did not yield any significant effects (*F* < 1; see Table 1 for all average RTs). Similarly, no effect of color scheme was observed in the results of a similar ANOVA on the accuracy data, *F*(2, 62) = 1.687, *p* = .194, η_p_^2^ = .052 (see Table 1 for all average accuracy scores). Importantly, overall mean accuracy was 82.93%, which was significantly better than chance level accuracy, (25%, one-sample *t* test), *t*(31) = 38.91, *p* < .001, confirming that participants had adequately learned the priority schemes.

**Table 1:** Mean correct response times (RT) and accuracy scores (ACC) for each color scheme (training and testing) in Experiment 1

Training Task		Scheme A		Scheme B		Scheme C	
	RT	897 (15)		911 (14)		910 (12)	
	ACC	83.71 (2.06)		83.70 (1.58)		81.39 (1.62)	
Testing Task		Scheme A		Scheme B		Scheme C	
	Shape Priority	T	D	T	D	T	D
	RT	953 (16)	946 (14)	942 (13)	949 (18)	943 (14)	953 (14)
	ACC	86.02 (2.18)	84.69 (2.19)	84.61 (2.41)	86.92 (2.18)	86.33 (2.33)	85.91 (2.08)

*Note.* In the testing task, the numbers under the “T” columns represent average performance on trials in which the target was presented in a prioritized color, compared with numbers under the “D” columns, which reflect performance on trials in which the distractor was presented in a prioritized color (i.e., shape priority). The numbers in brackets represent 95% within-subjects confidence intervals (Cousineau, 2005; Morey, 2008)

#### Priority testing task

As every trial in the testing task included two colors from the training task, we compared performance in trials with the target shape presented in a prioritized color to performance in trials with the distractor shape presented in the prioritized color. Practice and erroneous trials (16.49% of total trials) were excluded from the response-time analyses and the resultant data were also trimmed to normality by removing trials with response times that were 2.5 standard deviations above the conditional mean or faster than 200 ms (calculated separately for each subject and color scheme; 0.95% of total trials discarded). A repeated-measures ANOVA on the mean response times, with color scheme (Scheme A, B, or C) and shape priority (target, distractor; was the target or the distractor presented in the higher of the two prioritized colors) as within-subject factors revealed neither evidence of effects of color scheme (*F* < 1) or shape priority (*F* < 1), nor an interaction between these factors (*F* < 1; see Table 1 for all average RTs). Despite not observing an interaction between color scheme and shape priority, a series of paired-samples *t* tests were conducted to gauge whether difference between target and distractor priority were present for any of the color schemes. However, no such effects were observed (all *p* values > .428).

The same patterns of null effects were observed when the ANOVA was carried out on the accuracy data, shape priority: *F* < 1; color scheme: *F* < 1; Shape Priority × Color Scheme: *F*(2, 62) = 1.169, *p* = .318, η_p_^2^ = .036. Again, no differences between target and distractor priority were present for any of the color schemes (all *p* values > .207). Table 1 details the average response times, accuracy, and corresponding measures of variance for each of the three schemes in both the training and testing tasks.

### Discussion

The results of Experiment 1 provided no evidence that differentially prioritized stimuli have differing effects on attentional capture when presented simultaneously. No differences in response times were observed between trials with the target versus distractor shape presented in a prioritized color. Importantly, priority was only established at the trial level in Experiment 1 and averaged out over the three possible colors throughout the course of the training task. Although these patterns of null effects are not conclusive, they suggest that this sort of situational prioritization at the trial level may not elicit differential effects of selection history. We tested prioritization at the trial level, but not the task level, as a proxy for situational priority relationships in the real world where a given stimulus or feature may be prioritized in one situation but deprioritized in another situation. Although Experiment 1 did not uncover any evidence that this sort of situational prioritization takes places at the task level, we tested the possibility that stimuli with fixed priority relationships throughout the course of both training and testing tasks may elicit effects of selection history in Experiment 2.

## Experiment 2

In Experiment 2, we tested whether consistently prioritizing one color over another would lead to attentional capture by stimuli that were prioritized at the trial *and* the task level (as in any reward study; see Anderson et al., 2011b; Anderson & Yantis, 2013). The general methods of Experiment 2 were similar to those used in Experiment 1, with one important modification. Whereas prioritization of a given color averaged out over all trials in Experiment 1, in Experiment 2, one color was always given the highest priority (HPC) over the other two colors, the second color was always given low priority (LPC), and the third color was never given priority (NPC) throughout all trials in the training task, such that observers learned fixed priority relationships between the three colors at both the trial and task levels. Therefore, any selection history effects observed in the testing task of Experiment 2 in terms of faster response times for targets versus distractors presented in the color consistently prioritized in the training task can be attributed to the fixed priority relationships established between the three colors at the trial and task levels during the training task.

### Method

#### Participants

We tested 30 participants with normal or corrected-to-normal vision and no history of mental illness. Two participants (not included in these demographics) were replaced due to technical difficulties during experimentation. Of the remaining 28 participants, 21 were female, with an overall participant age of 21.54 years (*SD* = 4.76 years).

##### Training task

The training stimuli and procedure used in Experiment 2 were the same as in Experiment 1, except for the fixed versus situational priority relationships established between the three colors in the training task. An example of one possible fixed priority scheme is illustrated in the right panel of Fig. 1. Here, red is the high priority color (HPC), such that when a red stimulus is presented in the training task displays, it always contains the target T regardless of which of the other two colors is also present in the display (Schemes A & B). In this example, green is the low priority color (LPC), and only signals the target location when presented in the same display as a blue shape (Scheme C). Blue is the no priority color (NPC) and never contains the target T regardless of whether it is presented inside a red or green shape. The specific pairings of the three colors and priority levels for HPC, LPC, and NPC colors were counterbalanced over participants. To ensure that the frequency of selecting targets in a given color would not confound any fixed priority effects, the target was presented in the HPC and LPC equally often by using Schemes A and B on a random 25% of trials and Scheme C in the remaining 50% of trials. The whole experiment consisted of six blocks of 96 trials each, preceded by two practice blocks of 64 trials each.

##### Testing task

The priority testing task was also similar to the training task in terms of stimulus presentation and timing, with a few crucial differences. As in Experiment 1, the stimulus containing the target T was defined as the singleton shape present in the testing display (i.e., a diamond among circles, or a circle among diamonds), meaning color was no longer prioritized by target selection. However, in Experiment 2, only one colored stimulus was present in the testing display on a random 50% of trials (192 trials), and two colored stimuli were present on the other 50% of testing trials. When only one colored stimulus was presented, it was never the singleton shape target and instead functioned as a pop-out distractor. When two colored stimuli were presented, one was always the target singleton shape, and the other was the salient distractor shape. We included trials with only one color to measure the extent to which attention was captured by colored stimuli with different priorities, independent of the learned color-response contingencies between pairs of colors. We used the trials with two colors to compare how stimuli with fixed priority relationships compete for attentional resources. Every color pair was presented equally often, such that the target was presented in a higher priority color (HPC or LPC) than the distractor (LPC or NPC) on a random 50% of these two-color trials in the testing task and the distractor was presented in the higher priority color compared to the target in the remaining half of testing trials. The testing phase consisted of four blocks of 96 trials (384 trials in total), preceded by a single practice block of 30 trials.

### Results

#### Training task

To determine whether the three different priority schemes affected response times in the training task, we first conducted a repeated-measures ANOVA on participants’ nonpractice, correct (5.62% of total trials discarded), 2.5-standard-deviation-normalized response times (calculated separately for each subject and condition; 2.32% of the remaining trials discarded). There was a main effect of scheme, *F*(2, 54) = 23.004, *p <* .001, η_p_^2^ = .460, such that follow-up planned comparisons confirmed that correct response times for Scheme B trials were significantly faster (641 ms) than response times for Scheme A trials (690 ms, *p* < .001) and Scheme C trials (685 ms, *p* < .001), but that response times for Scheme A and Scheme C trials were not significantly different (*p* = .589). Overall accuracy scores were relatively high (94.70% correct) and well above 25% chance performance, *t*(27) = 131.833, *p* < .001. A second repeated-measures ANOVA on the accuracy data also revealed a main effect of scheme on accuracy (Scheme A: 92.10%; Scheme B: 96.68%; Scheme C: 95.31%), *F*(2, 54) = 20.077, *p* < .001, η_p_^2^ = .426. Follow-up planned *t* tests explicated that mean accuracy for trials in each of the three schemes differed significantly, with the most accurate performance occurring for Scheme B (Scheme B: 96.68%, Scheme C: 95.31%, Scheme A: 92.10%: largest *p* = .026). See Table 2 for all reaction times and accuracy means for the training and testing tasks.

**Table 2:** Mean correct response times (RT) and mean accuracy scores for each color scheme and overall for each task (training and testing) in Experiment 2

Training Task			Scheme A		Scheme B			Scheme C		
	RT		690 (22)		641 (14)			685 (20)		
	ACC		92.10(1.07)		96.68 (0.70)			95.31 (0.92)		
		One Color			Two Color					
Testing Task		HPC	LPC	NPC	Scheme A		Scheme B		Scheme C	
	Shape Priority	D	D	D	T	D	T	D	T	D
	RT	806 (6)	792 (6)	797 (7)	837 (12)	867 (18)	822 (17)	857 (14)	822 (11)	876 (14)
	ACC	86.74 (5.50)	90.04 (4.99)	89.98 (5.14)	83.01 (2.94)	81.76 (2.61)	85.67 (2.20)	84.08 (2.54)	86.44 (1.90)	79.96 (3.24)

*Note.* The numbers in brackets represent 95% within-subjects confidence intervals (Cousineau, 2005; Morey, 2008). In the testing task, the numbers under the “T” columns represent average performance in trials with the target presented in the training task prioritized color, compared with numbers under the “D” columns, which represent performance in trials with the distractor presented in the training task prioritized color (i.e., shape priority)

#### Testing task

**Fig. 3:**
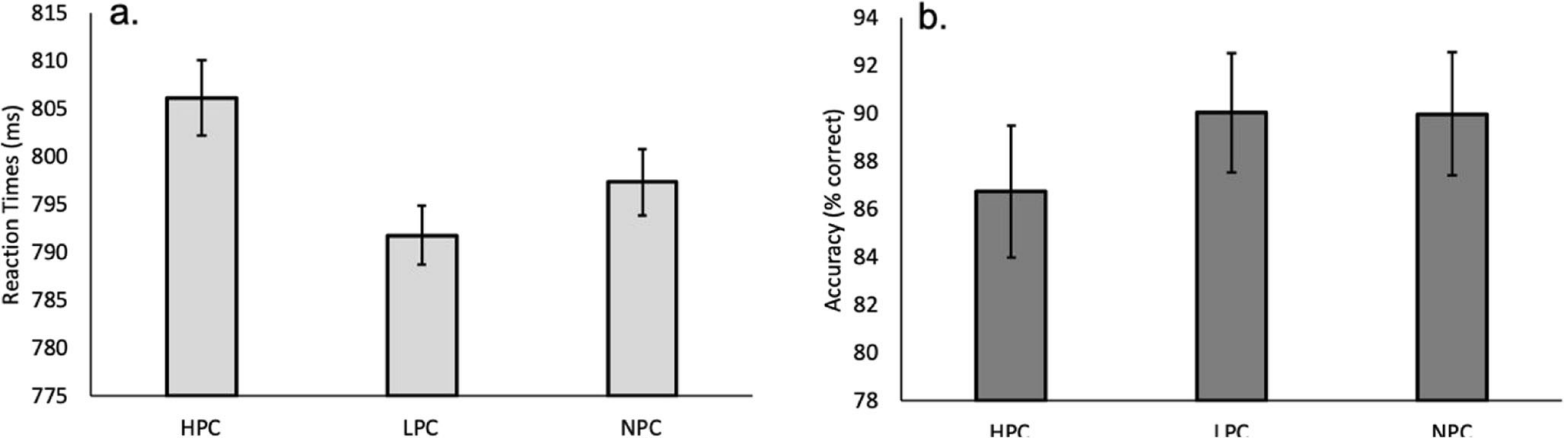
Mean correct reaction times (**a**) and mean accuracy scores (**b**) for the testing task for one color (distractor) trials: Correct response times to an uncolored shape singleton target in the testing task as a function of the three possible colored distractor priorities learned in the training task. Participants made slower correct responses to targets when a high-priority color (HPC) distractor was present in the search display compared with trials with a low-priority color (LPC) or nonpriority color (NPC) distractor. Similarly, participants responded the least accurately on trials in which the distractor was presented in the high-priority color. Error bars reflect 95% confidence intervals, corrected for within-subject designs (Cousineau, 2005; Morey, 2008)

**Fig. 4:**
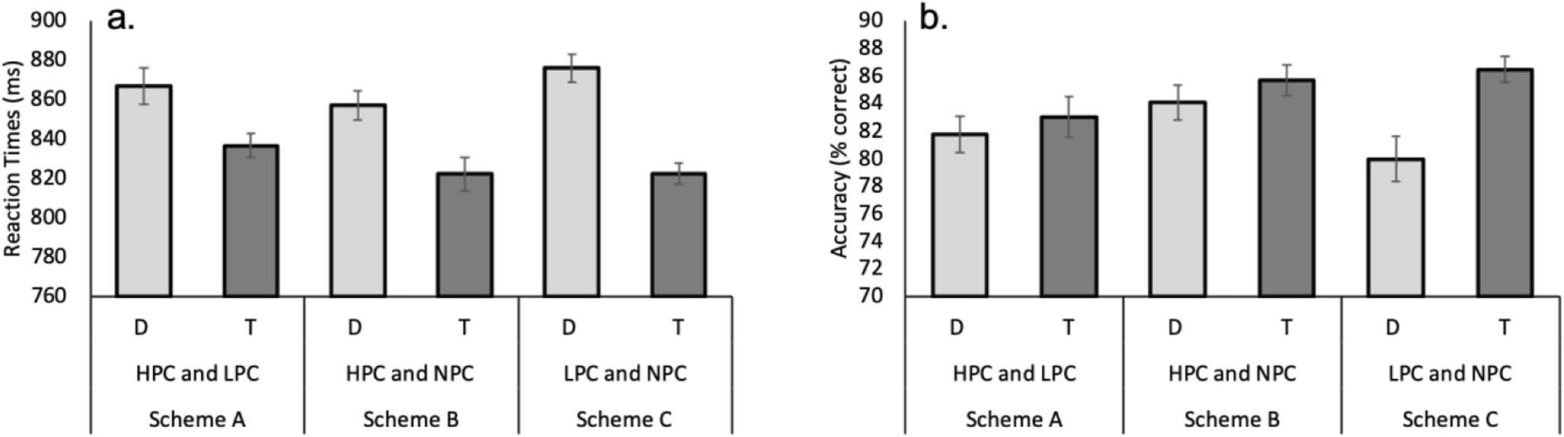
Mean correct reaction times (**a**) and mean accuracy scores (**b**) for the testing task for two-color (target, distractor) trials: Correct response times for trials with distractors or targets presented in the higher priority color as a function of the three priority schemes in the testing task of Experiment 2. Overall, participants responded faster in trials with the target presented in the higher priority color compared with their average correct response times in trials with the distractor presented in the higher priority color. Similarly, participants responded more accurately when the target was presenter in the higher priority color. Error bars reflect 95% confidence intervals, corrected for within-subject designs (Cousineau, 2005; Morey, 2008)

After confirming that fixed priorities were successfully associated with the three colors in the training task, we next analyzed the testing task data to determine whether the color priorities instilled at the trial and task levels in the training task would carry over to capture attention in the testing task. As in all previous analyses, we only analyzed nonpractice trial correct responses (15.95% of total trials discarded) within 2.5 standard deviations above the participant’s conditional mean (1.86% of remaining trials discarded). We then conducted separate analyses on response time and accuracy data for trials with only one color (distractor; see Fig. 3) and trials with two colors (target and distractor; see Fig. 4).

A repeated-measures ANOVA on participants’ averaged response times for one priority color (distractor) trials revealed a significant main effect of this color (HPC: 806 ms; LPC: 792 ms; NPC: 797 ms), *F*(2, 54) = 3.233, *p* = .047, η_p_^2^ = .107 (see Fig. 3a and Table 2). Planned comparisons confirmed that participants were slowest to respond to the target stimulus (i.e., the noncolored shape singleton) when a high-priority color distractor was present in the display compared with when a low-priority color distractor was present (*p* = .019). Surprisingly, the difference between high priority and no priority color failed to reach significance (*p* = .17). There was no significant difference in response times between trials with low and no priority color distractors (*p* = .26). Accuracy results were in line with the reaction time observations, as indicated by a main effect of priority (HPC: 86.74%; LPC: 90.04% ms; NPC: 89.98%), *F*(2, 54) = 5.254, *p* = .008, η_p_^2^ = .163 (see Fig. 3b and Table 2). Planned *t* tests showed that participants were significantly less accurate on trials with a high-priority distractor compared with trials with a low-priority (*p <* .001) or no-priority distractor (*p* = .018). No difference was observed between trials with a low versus no-priority distractor (*p* = .96).

To investigate whether color prioritization in the training task carried over in the form of lingering attentional bias in the testing task, we next conducted a repeated-measures ANOVA, with shape priority (target, distractor) and scheme (A, B, C) on participants’ averaged response times in the two-color trials. Importantly, each display in the two-color trials always consisted of one shape presented in a color that previously had a higher priority than the color of the other shape. On half the trials, the target shape singleton was presented in the higher priority color and the distractor in the lower priority color, and vice versa on the remaining trials. The ANOVA resulted in a main effect of shape priority, *F*(2, 54) = 29.102, *p* < .001, η_p_^2^ = .519, such that participants responded faster to trials in which target was prioritized over the distractor (827 ms) compared with response times for trials in which the distractor was prioritized over the target (866 ms; see Fig. 4a and Table 2). Although there was neither a significant main effect of scheme, *F*(2, 54) = 1.520, *p =* .228, η_p_^2^ = .053, nor a significant interaction, *F*(2, 54) = 1.314, *p =* .277, η_p_^2^ = .046, we nevertheless conducted planned comparisons between the three schemes to quantify any lingering effects of differential prioritization. These comparisons revealed significantly faster correct response times for trials with targets versus distractors presented in the prioritized color for each of the three scheme (Δ Scheme A: 30 ms; *p* = .018; Δ Scheme B: 35 ms; *p* = .009; Δ Scheme C: 54 ms; *p* < .001), suggesting that imbalances in prioritization carry over as lingering effects of selection history.

Finally, a repeated-measures ANOVA on the accuracy data, with shape priority (target, distractor) and scheme (A, B, C), revealed a main effect of shape priority, indicating that participants responded more accurately when the target was prioritized over the distractor (85.0% correct) as compared with trials in which the distractor was prioritized over the target (81.9% correct), *F*(1, 27) = 7.438, *p* = .011, η_p_^2^ = .216. No significant effect of scheme was observed, *F*(1, 27) = 1.739, *p* = .185, η_p_^2^ = .061, nor did the interaction between scheme and shape priority reach significance, *F*(2, 54) = 2.520, *p* = .090, η_p_^2^ = .085 (see Fig. 4b and Table 2).

### Discussion

Experiment 2 confirmed that effects of selection history can be observed when stimuli are prioritized in a fixed manner at both the task and trial levels. Effects of prioritization were already apparent in the training task, where participants responded faster and more accurately for Scheme B trials compared with Scheme A or C trials. Although these results could potentially be caused by selection history effects, they are at least equally likely caused by differences in ambiguity concerning which stimulus a participant has to respond to. In Scheme B, there is the least ambiguity about which stimulus contains the target because the target was always presented in the high-priority color (the color that always required a response) and the distractor was always presented in the no-priority color (the color that never required a response). Given that high overall accuracy in the training task suggested the color schemes were all well learned (like in Experiment 1), we instead turned to the testing data to further quantify the effects of fixed prioritization. The testing phase data showed clear lingering effects due to selection history, such that a stimulus with a higher versus lower priority had more influence on attentional allocation. This pattern was observed for all paired levels of prioritization (HPC, LPC, and NPC). Therefore, the results of Experiment 2 suggest that fixed prioritization influences attention even when the prioritized feature is no longer task relevant.

### Cross-experiment analysis

We also conducted a cross-experiment mixed-design ANOVA, with shape priority as a within-subjects factor and experiment as a between-subjects factor on the averaged response-time data for trials in both experiments with two colored stimuli, to assess potential differences between selection effects due to situational (Experiment 1) versus fixed (Experiment 2) prioritization. We observed a main effect of shape priority, such that participants made faster correct responses when the shape singleton target was presented in the higher priority color (887 ms) compared with correct response times for trials with the distractor presented in the higher priority color (908 ms), *F*(1, 58) = 20.227, *p <* .001, η_p_^2^ = .259. As expected, there was a significant interaction between shape priority and experiment, *F*(1, 58) = 15.158, *p =* .001, η_p_^2^ = .207. Follow-up planned comparisons showed no significant difference between target and distractor prioritization in Experiment 1 (Δ 3 ms, *p* = .641), but a significant difference between target and distractor priority in Experiment 2 (Δ 40 ms, *p* < .001), confirming that the main effect of shape priority was driven by the fixed priority relationships established in Experiment 2*.* A similar interaction between shape priority and experiment was observed for the accuracy scores, indicating that the difference between target and distractor priority in Experiment 2 (3.11%, *p* = .011), was larger than the same effect in Experiment 1 (Δ 0.1%, *p* = .824). These results once more confirm that situational priority does not lead to observable differences in terms of selection history, whereas fixed priority does.

## General discussion

We investigated whether stimuli prioritized during an initial training task would lead to attentional biases due to selection history in a subsequent testing task where priority was no longer enforced or task relevant. Indeed, results suggest that previously prioritized stimuli can lead to attentional capture. However, whether capture occurs depends on the nature of the priority relationships. Significant effects of attentional capture by previously prioritized stimuli were observed only in Experiment 2, when the prioritized stimuli shared a fixed priority relationship such that throughout the experiment, different colors had a ranked priority that never changed. Experiment 2 showed that under such fixed circumstances, when a single colored stimulus was presented as one of the distractors in the testing task, attentional capture was observed such that correct response times were slower and accuracy decreased when the distractor was presented in the HPC compared with when it was presented in the LPC. When both the target and distractor were presented in prioritized colors, response times were biased by the higher priority stimulus such that participants were faster when the target versus distractor was presented in the higher priority color. On the contrary, when stimuli were only situationally prioritized at the trial level in Experiment 1, there were no corresponding effects of capture during the testing phase. Furthermore, the significant interaction between shape priority and experiment in the cross-experiment analysis offered further support for the proposal that fixed, but not situational, priority induced lingering effects of selection history.

The present findings add to our understanding of the attentional phenomenon of selection history. A number of attentional effects have been retroactively linked to selection history, such as intertrial priming (Maljkovic & Nakayama, 1994, 1996; Theeuwes & Van der Burg, 2008, 2013) and statistical learning (Ferrante et al., 2018; Wang & Theeuwes, 2018). Furthermore, results of other studies can retrospectively be attributed to priority learning. For example, in a recent study by Feldmann-Wüstefeld, Uengoer, and Schubö (2015), participants performed an associative learning task in which two singleton stimuli were presented, one based on color and one based on shape. Half the participants were instructed to respond to the shape singleton, thereby potentially prioritizing shape over color, whereas the other half responded to the color singleton, thereby likely prioritizing color over shape. In a subsequent search task, participants had to search for a shape singleton, while intermittently a color distractor singleton was presented. This color distractor impaired search times to a much greater extent for participants who had prioritized color in the associative learning task. While the study by Feldmann-Wüstefeld and colleagues does not provide an explanation in terms of learned priority, this may very well be the underlying mechanism responsible for the observed results. Additionally, a study by Sali, Anderson, and Yantis (2014) describes a series of training/testing-based value-driven capture experiments. The main result of this study suggests that value-based capture only occurs for stimulus features that carry uniquely predictive information about reward. When no such information is available, no value-driven capture takes place. The study by Sali and colleagues can be interpreted in terms of prioritization: If stimulus features are randomly and inconsistently associated with reward, then the participant cannot prioritize one stimulus over another, and hence no capture takes place.

The current study is among the first nonreward studies to directly manipulate selection history through behavioral manipulation. By using independent training and testing tasks, we ensured complete independence between the development of a task-relevant top-down set and its subsequent effects on attentional selection when it was no longer task-relevant. Along these lines, our results also provide convincing evidence that selection history is not merely the result of prior attentional selection of certain stimuli or features, but that particular rules govern when prior selection will carry over in the form of subsequent attentional biases. The nature of the relationship between differently prioritized stimuli is key. Additional factors that operate throughout the training task, such as the fixed priority relationships established in Experiment 2, are necessary to elicit lingering effects indexed by attentional capture.

The current results suggest that effects of selection history evoked by learned prioritization can strongly modulate the priority map (Awh et al., 2012). The results of Experiment 2 show that stimuli that are overall more prioritized than other stimuli will most strongly modulate activation on the priority map, which leads to attentional biases toward these high-priority stimuli. The lack of attentional capture by the higher of the two prioritized colors in Experiment 1 appears to indicate that the map is based on overall relatively stable (i.e., fixed) priority relationships learned over time, rather than on individual situational relationships between stimuli. In other words, selection history-based activation on the priority map is not defined as the relative difference in priority between two stimuli presented simultaneously (i.e., priority defined at the trial level). This is evidenced by the absence of any differential lingering differences in the testing phase following situational priority learning (Experiment 1). That is not to say that situationally prioritized stimuli do not elicit increased activation on the priority map. Rather, any effects of selection history due to situational priority are fully negated by the tasks fixed priority levels. As these levels are equal for each stimulus in Experiment 1, their influences on the priority map must also be equal, and hence no biases in attentional modulation are observed. Overall, the present results provide a further step toward increasing our understanding of which factors guide attentional biases due to selection history, as well as defining the limits of selection history’s influence on attention.

Some criticism of the current work is warranted. First, although our results support the proposal that fixed priority relationships yield lingering effects of selection history, several methodological aspects limit the extent to which the results can be interpreted as absolute effects of selection history. For example, in both experiments, the colored stimuli always “popped out” compared with the other gray stimuli in the displays. Therefore, this bottom-up attentional capture likely restricted the competition for attentional resources to the two colored stimuli. Nonetheless, neither bottom-up nor top-down effects can fully explain the obtained results in Experiment 2, suggesting that, despite these potential limiting factors, selection history must be the driving mechanism behind the observed effects. Second, the current study uses an arbitrarily chosen cutoff for participant inclusion/exclusion (here, set as an accuracy score of 2.5 standard deviations below the group mean), which led to the exclusion of three participants in Experiment 1. Alternatively, we could have chosen to use a less strict criterion and exclude only those participants that scored below chance level. Using a chance-level criterion would not have led to the exclusion of the three participants in Experiment 1. Nonetheless, a recalculation of all statistical tests that included the data of the three discarded participants did not change any result and conclusion drawn here.

Finally, throughout the current study, theoretical comparisons between learned prioritization and value-driven attentional capture have been put forward. The current study provides support for recent studies that have shown that the administration of reward is not a necessity for observing lingering effects of stimulus history on attentional selection (Grubb & Li, 2018; Miranda & Palmer, 2014; Sha & Jiang, 2016; cf. Anderson & Halpern, 2017). For example, using a similar design as Anderson et al. (2011a, b), Sha and Jiang (2016) showed that after an initial training test without reward, reaction times to a shape singleton were significantly slower when a previously selected but unrewarded color stimulus was present as a distractor in the search display as compared with reaction times on trials in which this colored stimulus was absent, mirroring the effects of value-driven attentional capture tasks without administering any rewards. These studies (Grubb & Li, 2018; Miranda & Palmer, 2014; Sha & Jiang, 2016) provide tentative evidence that observed value-driven attentional capture effects may not necessarily be fully attributed to a lingering reward signal upon which selective attention acts (but see Anderson 2011b; Anderson & Halpern, 2018). Instead, the mere repeated selection of feature-defined targets appears to be sufficient for attention to be captured by these stimuli, even when the target-defining features are no longer task relevant.

Grubb and Li (2018) further propose that differences in attentional modulation by stimuli that previously signaled high versus low reward may also be caused by factors other than reward. According to Grubb and Li, observers could prioritize high rewards over low rewards during the training phase to maximize monetary gain (see also Le Pelley et al., 2016). As such, stimuli that are otherwise equally often selected and that are equally task relevant in the training task get unevenly prioritized by the observer, resulting in unequal lingering attentional biases in the testing task. The current study supports this nonreward interpretation by showing that differences in the speed of attentional selection between high and low are not necessarily caused by an increased reward signal, but rather may reflect an indirect shift in the way stimuli are differentially prioritized. While more work needs to be conducted to distinguish between prioritization and reward as the driving factors behind selection history based attentional modulation, the current study shows that prioritization may play a crucial role in selection history, and that certain results obtained in value-driven capture studies may need to be reevaluated in the context of prioritization.
